# Brain basis of cognitive resilience: Prefrontal cortex predicts better reading comprehension in relation to decoding

**DOI:** 10.1371/journal.pone.0198791

**Published:** 2018-06-14

**Authors:** Smadar Z. Patael, Emily A. Farris, Jessica M. Black, Roeland Hancock, John D. E. Gabrieli, Laurie E. Cutting, Fumiko Hoeft

**Affiliations:** 1 Department of Psychiatry and Weill Institute for Neurosciences, University of California, San Francisco, San Francisco, California, United States of America; 2 Department of Communication Disorders, Tel Aviv University, Tel Aviv, Israel; 3 Tennessee Center for the Study and Treatment of Dyslexia, Middle Tennessee State University, Murfreesboro, Tennessee, United States of America; 4 School of Social Work, McGuinn Hall, Boston College, Chestnut Hill, Massachusetts, United States of America; 5 Department of Psychological Sciences, University of Connecticut, Storrs, Connecticut, United States of America; 6 Brain Imaging Research Center, University of Connecticut, Storrs, Connecticut, United States of America; 7 Department of Brain and Cognitive Sciences and McGovern Institute for Brain Research, Massachusetts Institute of Technology, Cambridge, Massachusetts, United States of America; 8 Institute for Medical Engineering & Science, Cambridge, Massachusetts, United States of America; 9 Peabody College, Vanderbilt University, Nashville, Tennessee, United States of America; 10 Vanderbilt Brain Institute, Vanderbilt University, Nashville, Tennessee, United States of America; 11 Haskins Laboratories, New Haven, Connecticut, United States of America; 12 UC-Stanford Multi-University Precision Learning Center, San Francisco, California, United States of America; 13 Department of Neuropsychiatry, Keio University School of Medicine, Tokyo, Japan; Katholieke Universiteit Leuven, BELGIUM

## Abstract

**Objective:**

The ultimate goal of reading is to understand written text. To accomplish this, children must first master decoding, the ability to translate printed words into sounds. Although decoding and reading comprehension are highly interdependent, some children struggle to decode but comprehend well, whereas others with good decoding skills fail to comprehend. The neural basis underlying individual differences in this discrepancy between decoding and comprehension abilities is virtually unknown.

**Methods:**

We investigated the neural basis underlying reading discrepancy, defined as the difference between reading comprehension and decoding skills, in a three-part study: 1) The neuroanatomical basis of reading discrepancy in a cross-sectional sample of school-age children with a wide range of reading abilities (**Experiment-1**; n = 55); 2) Whether a discrepancy-related neural signature is present in beginning readers and predictive of future discrepancy (**Experiment-2**; n = 43); and 3) Whether discrepancy-related regions are part of a domain-general or a language specialized network, utilizing the 1000 Functional Connectome data and large-scale reverse inference from Neurosynth.org (**Experiment-3**).

**Results:**

Results converged onto the left dorsolateral prefrontal cortex (DLPFC), as related to having discrepantly higher reading comprehension relative to decoding ability. Increased gray matter volume (GMV) was associated with greater discrepancy (**Experiment-1**). Region-of-interest (ROI) analyses based on the left DLPFC cluster identified in **Experiment-1** revealed that regional GMV within this ROI in beginning readers predicted discrepancy three years later (**Experiment-2**). This region was associated with the fronto-parietal network that is considered fundamental for working memory and cognitive control (**Experiment-3**).

**Interpretation:**

Processes related to the prefrontal cortex might be linked to reading discrepancy. The findings may be important for understanding cognitive resilience, which we operationalize as those individuals with greater higher-order reading skills such as reading comprehension compared to lower-order reading skills such as decoding skills. Our study provides insights into reading development, existing theories of reading, and cognitive processes that are potentially significant to a wide range of reading disorders.

## Introduction

Becoming proficient in reading comprehension relies on mastering decoding [1], a skill that enables a child to map letters to their corresponding speech sounds and meaning. Indeed, seminal frameworks in reading and its development indicate that reading comprehension is highly dependent on a reader’s ability to decode words accurately, fluently and effortlessly [2]. Neuroimaging studies indicated that reading comprehension and decoding activated overlapping regions [3, 4]. Yet, there are exceptions. Some readers show a discrepancy between reading comprehension and decoding skills. While the term “discrepancy” has often been used to reflect differences between cognitive abilities (or aptitude such as IQ) and reading skills arising from a historical definition of dyslexia using the “discrepancy criteria” [5, 6], these readers have also been known as *discrepant readers*. While potentially confusing, for the purpose of consistency, we use the term discrepant readers to describe individuals with large differences between reading comprehension and decoding with possible discrepancy in either direction (either comprehension better than decoding or decoding better than comprehension). There are two profiles that anchor the spectrum of discrepant readers: those who have low decoding, but relatively good comprehension skills, known as *resilient dyslexia* [7–10], and those who have low comprehension skills but relative good decoding known as *specific reading comprehension disorder*, *S-RCD* [11–14]. The aim of this study was twofold; first, to characterize the neurobiological structural of discrepant readers in children with a wide range of reading abilities; and second, to examine in an independent dataset whether the brain regions associated with reading discrepancy are a consequence of learned compensatory strategies that develop over time or whether these regions are in place prior to reading acquisition and predictive of later discrepancy.

Although decoding and comprehension skills are typically strongly related, some readers exhibit a significant discrepancy between these skills. These discrepant readers drew the attention of researchers and educators since they highlight different mechanisms that potentially influence these skills. To date, most of the studies that examined factors that moderate the decoding-comprehension relationship are based on the assumptions made by the *Simple View of Reading* model [15, 16]. According to this model, reading comprehension is the product of two components—decoding and oral language comprehension. Thus, according to this model, oral language comprehension is what accounts for the discrepancy in the two profiles of discrepant readers. On one end of the reading discrepancy spectrum are readers with specific reading comprehension disorder (S-RCD), who struggle with reading comprehension despite adequate decoding. This reading profile is observed in approximately 10% of school-aged children [13, 14, 17] and is strongly related to poor oral language skills in school [18, 19] and to a history of language impairment in preschool or kindergarten [20, 21]. A contrasting profile is the resilient dyslexics, who are readers with developmental dyslexia that, despite their poor decoding skills, comprehend text relatively well [7–10]. Such conditions have been observed in about 3% of a representative sample of university students [7]. This discrepancy has been attributed to good oral language skills, in particular, strong semantic skills [8].

Increasing evidence has garnered empirical findings for the unique contribution of non-linguistic skills, such as cognitive control and working memory to reading comprehension [4, 22–26], providing support for the inclusion of these skills in a theoretical model of reading comprehension [4]. Studies have shown that skills such planning, shifting and, working memory have been moderately associated with reading comprehension, above and beyond skills ascribed in the Simple View of Reading model [4, 24]. Moreover, S-RCD show poor performance in planning, organization, and working memory tasks [14, 26–28]. On the other hand, good performance in these skills have been found to play a compensatory role for children with low decoding skills [29]. These skills of cognitive control and working memory may enable readers to develop and revise plans for reading a text, making inferences by integrating incoming information with prior knowledge as well as previously read text information, and inhibit ideas or information not textually relevant during reading [24, 30–32].

Sparse neuroimaging evidence limits a cohesive and comprehensive understanding of the neural architecture that supports such reading discrepancies. Neuroimaging studies indicate that reading comprehension and decoding activate overlapping regions, yet comprehension elicits a broader network that supports language and cognitive control [3, 4, 33]. Moreover, a handful of studies that examined discrepant readers show associations between reading discrepancy and right inferior frontal regions [17, 34, 35]. One study found adult resilient dyslexics and proficient readers to show greater radial expansion in the right inferior frontal region, which is a measure of local brain size. This finding was attributed to reflect good comprehension [34]. Two studies using structural and functional MRI comparing S-RCD, dyslexic readers, and proficient readers indicated that readers with S-RCD might have a distinct profile from the dyslexic readers and proficient readers (15, 33). Occipito-temporal region and the supramarginal gyrus, which have been associated with orthographic and phonological processing, were found to differentiate between the dyslexic group and the other groups that included S-RCD and proficient readers. However, the S-RCD group compared to the other groups showed additional reduced gray matter in the right frontal areas and anomalies in connectivity between the left inferior frontal gyrus when reading low versus high frequency words. These regions are known to be important for semantic processing and executive functions. Taken together, these studies reflect behavioral findings of resilient dyslexia and S-RCD, which suggest that whereas resilient dyslexics showed only anomalies in regions related to processing of phonological and orthographic forms, the S-RCD showed anomalies in regions related to semantic processing and executive functions. However, no study has examined the neurobiological structure of discrepant readers along the entire continuum from poor to typical decoding. Collectively, results from behavioral studies that encompass discrepancy in decoding and reading comprehension have not been consistent in whether language or cognitive measures contribute to this discrepancy. This is possibly due to differences in sample composition among other possible factors. Furthermore, it is unknown whether anomalies in these regions precede or follow the development of this discrepancy as children learn to read. That is, it is unknown if the findings are a secondary consequence of the enhanced use of compensatory strategies. Past studies were done in older children and adults, already with years of formal reading instruction. Studies in beginning readers that track the development of discrepancy is needed.

In the current study, we therefore investigated the brain basis underlying discrepancy between decoding and reading comprehension. We first examined the associations between reading discrepancy and gray matter volume (GMV) in school-age children with a wide range of reading abilities (**Experiment-1**; n = 55). We hypothesized that if discrepancy is mediated by a domain-specific language network, as the Simple View of Reading suggests, key findings would converge in brain structures associated with language and oral comprehension [15, 16] such as the left inferior frontal and anterior temporal regions [36]. On the other hand, if discrepancy is mediated by domain-general networks associated with cognitive control and working memory [4, 22, 23], structures such as the dorsolateral prefrontal cortex (DLPFC) would be involved [17, 35, 37].

We then investigated whether the neuroanatomical region that we found associated with reading discrepancy, preceded or followed reading discrepancy and if it is secondary to literacy acquisition (**Experiment-2**, n = 43). We also examined whether these brain structures in beginning readers predict the development of discrepancy when they become proficient readers using regions identified in **Experiment-1**. Finally, to determine whether discrepancy-related regions are part of a domain-general network or a language network, we used a large-scale neuroimaging database (**Experiment-3**). This methodology offers the opportunity to perform comprehensive analyses of reverse inference to identify the psychological contributions of a given brain region. Thus, it is commonly used to decide among competing theories of human behavior [38, 39]. In the current study, we used the Neurosynth database that included the 1000 Functional Connectome Project [40] to analyze resting-state functional connectivity (rsFC) from the region identified as the discrepancy-related anatomical cluster found in **Experiment-1** as the seed. We then applied large-scale reverse inferences to the rsFC circuits.

## Materials and methods

### Experiment-1: Individual differences in the neural correlates of reading discrepancy in school-age children

#### Participants

We studied native English-speakers (n = 55; 27 females; 52 right-handed) between the ages of 10–16 years (13.9±2.1). A subgroup of 26 children (47.4% of the sample) had a formal diagnosis of reading disorders and scored below the 23th percentile rank in decoding or reading comprehension or both (see below for the description of these measures). Demographic, cognitive, language and reading performances are presented in **Table 1**. All participants had a nonverbal intelligence quotient (IQ) within one SD of the norm, as measured by the Wechsler Abbreviated Scale of Intelligence—Matrix Reasoning (WASI-MR) [41]. None of the participants had any parental reports of a formal diagnosis of neurological or psychiatric disorders, including attention-deficit/hyperactivity disorder, were on any current medication, or had contraindications to magnetic resonance imaging (MRI). However, as reading disorders, such as dyslexia and S-RCD, are highly co-occurring with developmental language disorders [18–20], we cannot rule out that some of the participants in the study, particularly those with a reading disorder, also had language disorders. Participants overlapped partially with individuals included in our previous studies [42–44]. The socioeconomic status of the participants’ families was weighted toward high SES. The Stanford University and University of California San Francisco Panels on Human Subjects in Medical Research approved the study and a written informed assent and consent was obtained from each child and guardian.

**Table 1:** Experiment-1: School-aged sample (*n* = 55). Demographic and behavioral performance and their correlations with the discrepancy index (DiscInd), decoding (DECODE) and reading comprehension skills (COMP).

	Mean (*SD*)	*r* with DiscInd	*r* with DECODE	*r* with COMP
Age	13.89 y (2.08)	-0.03	-0.17	-0.15
Gender	28 M/ 27 F	-0.10	-0.13	-0.17
Handedness	52 R/ 3 L	0.03	-0.09	-0.04
DiscInd [SS]	0.58 (12.35)		-0.11	0.67***
DECODE (WRMT-WA) [SS]	95.67 (12.43)			0.67***
COMP (WRMT-PC) [SS]	96.25 (16.54)			
WASI-MR [SS]	108.00 (11.04)	0.17	0.45***	0.47***
PPVT [SS]	98.96 (18.16)	0.54***	0.54***	0.81***
CTOPP-MD [SS] ^a^	100. 09 (17.79)	0.33*	0.54***	0.66***
GORT Rate [SS]	88.18 (17.03)	0.35**	0.60***	0.71***
GORT Fluency [SS]	86.76 (27.27)	0.30*	0.81***	0.85***
GORT-COMP [SS]	99.45 (17.26)	0.53***	0.56***	0.82***
TOWRE-PDE [SS]	88.71 (16.55)	0.15	0.92***	0.80***
TOWRE-SWE [SS]	91.2 (13.54)	0.44	0.68***	0.85***
RAN Average [SS]	102.93 (17.3)	0.35*	0.50**	0.65***
WJ-Spelling [SS] ^a^	91.41 (18.28)	0.22	0.78***	0.76***
WJ-Writing Fluency [SS] ^a^	99.26 (15.01)	0.53***	0.49***	0.77***
Total GMV	693.6 (61.29)	-0.05	-0.11	-0.12

DiscInd: Discrepancy index, WRMT-PC minus WRMT-PC; DECODE: Woodcock Reading Mastery Test Word Attack Subtest (WRMT-WA); COMP: Woodcock Reading Mastery Test Revised Passage Comprehension Subtest (WRMT-PC);WASI-MR: Wechsler Abbreviated Scale of Intelligence Matrix Reasoning Subtest; PPVT: Peabody Picture Vocabulary Test-3; Fluency composite: average of TOWRE-SWE: Tests of Word Reading Efficiency 2 Sight Word Efficiency Subtest and GORT Fluency: Gray Oral Reading Tests fluency 3 Subset; CTOPP-MD: Comprehensive Test of Phonological Processing Memory for Digits Subtest; GORT Rate: Gray Oral Reading Tests 3 Rate Subset; GORT Fluency: Gray Oral Reading Tests 3 Fluency Subtest; GORT-COMP: Gray Oral Reading Tests 3 Comprehension Subtest; TOWRE-PDE: Tests of Word Reading Efficiency 2 Phonemic Decoding Efficiency Subtest; TOWRE-SWE: Tests of Word Reading Efficiency 2 Sight Word Efficiency Subtest; RAN Average: Average of Rapid Automatized Naming Color, Object, Letter and Number Subtests; WJ-Spelling: Woodcock-Johnson Spelling Subtest; WJ-Writing Fluency: Woodcock-Johnson Writing Fluency Subtest. GMV: Gray Matter Volume; [SS] standard score (norm = 100, SD = 15); y: years; M: male; F: female; R: right; L: left

*p < 0.05

** *p* < 0.01

*** *p* < 0.001.

^a^ n = 54

#### Neuropsychological measures

A standard battery of neuropsychological measures was obtained that included decoding, reading comprehension and IQ measures. Children’s decoding ability (DECODE) was assessed by Woodcock Reading Mastery Test Revised—Word Attack subtest (WRMT-WA) [45], which examines the subject's ability to phonetically decode nonwords. The children’s reading comprehension abilities (COMP) were assessed by the Passage Comprehension test, a subtest of the WRMT (WRMT-PC) [45], which requires reading of a single-sentence or a short passage and filling in the missing word in empty blanks (cloze format). The discrepancy between COMP and DECODE was computed by the difference between the COMP and DECODE measurements, i.e., the standard scores (SS) of WRMT-PC and WRMT-WA (Discrepancy Index, DiscInd). Our primary analysis examined discrepancy by using difference scores as the primary dependent variable of interest rather than regressing out DECODE from COMP, as has been done in other studies [46, 47]. We used a difference score because of its extensive use in past studies in different fields [34]. It is a widely-employed approach in clinical practice due to its simplicity, making our results more generalizable. It can be easily calculated without concerns of calculating residuals from a small sample (as was the case in our study) or estimating a function based on an independent large sample. Moreover, when we repeated the analyses by partialling out DECODE from COMP the results were unchanged (see below). Yet, as a result of how DiscInd was calculated (i.e., differences between DECODE and COMP scores), it was not possible to partial out DECODE or COMP from DiscInd. Therefore, we controlled for such potential confounds in preliminary analyses reported in the **S1 File**. These supporting analyses **effectively controlled for the potential confounds by** comparing resilient dyslexics (with poor decoding and discrepantly high reading comprehension), with two control groups of non-discrepant readers who were either matched for decoding skills (to control for DECODE), or matched for reading comprehension skills (to control for COMP) in the results found above (i.e., left DLPFC GMV).

Since participants can differ across reading comprehension measures in the cognitive constructs (e.g. they rely more on executive processes versus semantic processes) [14, 48], it was important to validate our findings across different reading comprehension tests. Thus, we repeated the analysis using a different discrepancy index based on the comprehension score of the Gray Oral Reading Test-3 (GORT-COMP) [49] instead of the WRMT-PC score. GORT-3 is a standardized reading comprehension test which requires reading expository and narrative passages between 85–150 words and answering multiple-choice questions. This DiscInd-GORT was calculated by subtracting the SS of DECODE from the SS of GORT-COMP, similar to how we calculated DiscInd with WRMT-PC. DiscInd and DiscInd-GORT were highly significantly correlated (*r* = 0.63, *p* < 0.001).

Additionally, possible confounding factors of nonverbal IQ and reading fluency were controlled. Nonverbal IQ was assessed by the WASI-MR [41]. Reading fluency was assessed by a subtest measure of rate from the GORT [49], which measures reading rate of a short paragraph with words embedded within context. This fluency measure was highly correlated with another fluency measure—the Sight Word Efficiency, a subtest of the Test of Word Reading Efficiency-2 (TOWRE-SWE) [50], which measures fluency of reading isolated words (*r* = 0.89, *p* < 0.001).

Additional assessments of language, working memory, reading, and spelling were acquired, including the Peabody Picture Vocabulary Test-3 (PPVT) [51], the Comprehensive Test of Phonological Processing Memory for Digits Subtest (CTOPP-MD) [52], the TOWRE—Phonemic the Decoding Efficiency subtest (TOWRE-PDE) [52], the Woodcock-Johnson III Spelling and writing fluency subtests [53], and the Rapid Automatized Naming (RAN) of Colors, Objects, Numbers and Letters [54]. Detailed participant characteristics are provided in **Table 1**.

#### MRI data acquisition and processing

Imaging-related procedures were performed at the Richard M. Lucas Center for Imaging at Stanford University using a 3 Tesla GE Signa LX scanner (GE, Milwaukee, WI USA) and a custom-built volume head coil. Three-dimensional high-resolution anatomical scans were acquired using a spoiled gradient echo (SPGR) pulse sequence (Echo time [TE] = 2ms, Repetition time [TR] = 9ms, Flip angle [FA] = 15°, number of excitations [NEX] = 2) that produced 124 coronal T1-weighted images with a field of view (FOV) of 24cm and voxel sizes of 0.94 x 0.94 x 1.2mm.

Image processing was performed using Diffeomorphic Anatomical Registration Through Exponentiated Lie Algebra (DARTEL) for voxel-based morphometry 8 (VBM8) toolboxes in Statistical Parametric Mapping 8 software (SPM8; Wellcome Department of Cognitive Neurology, London, UK; http://www.fil.ion.ucl.ac.uk/spm) [55]. Images were bias-field corrected and segmented to gray matter (GM), white matter (WM) and cerebro-spinal fluid (CSF). The images were spatially normalized to 1.5 x 1.5 x 1.5mm voxels in the Montreal Neurological Institute (MNI) stereotaxic space using nonlinear registration, modulated, and smoothed with an 8-mm isotropic Gaussian kernel. We selected the modulated, normalized, and non-linear only option, which resulted in an analysis of relative differences in regional GMV, corrected for individual brain size. Analysis was constrained to GM using a threshold masking of 0.3 on an averaged image of normalized and unsmoothed images from individual subjects. Voxel-based morphometry (VBM) [56] analyses of regional GMV were then conducted on a voxel-by-voxel basis.

#### Statistical analyses

To investigate whether regional GMV across the whole brain related to individual differences in decoding skills, reading comprehension skills and reading discrepancy, we used three multiple linear regression models relating voxel-wise GMV to DECODE, COMP and DiscInd, respectively. Age at time of scan and gender were included as covariates in the model to control for anatomical differences, as recommended for VBM studies [57].

Control analyses were performed in several ways. First, to assess discrepancy using different approaches, we assessed the correlation of GMV with a discrepancy residual score by partialling out DECODE from COMP using whole brain analysis. We also examined the effect of DECODE partialling out COMP. Second, to ensure the findings were not solely driven from the specific reading test we used, the basic analysis was repeated with DiscInd-GORT as a dependent measure using whole brain analysis. Third, the basic analysis with DiscInd as a dependent variable was repeated to examine the effect of GMV after controlling for other factors commonly associated with reading comprehension, including nonverbal IQ and reading fluency using whole brain analyses. Further, to eliminate the possibility that children with dyslexia are the only ones that account for the result, we tested whether there was an interaction between dyslexia diagnosis and regional GMV in association with reading discrepancy. For this analysis, we extracted mean GMV of the cluster related to DiscInd that we obtained from the whole brain analysis, by averaging voxel-wise GMV across all voxels within the region. Then, a regression analysis with DiscInd as the dependent variable was performed, including GROUP (dyslexia, typical readers), GMV of the region of interest (ROI), and GROUP × GMV interaction term as regressors. For all control analyses, age and gender were controlled and used as nuisance regressors.

A statistical threshold of *p* < 0.05 corrected for multiple comparisons was used, determined by Monte Carlo simulations in AFNI’s 3dClustSim [58], which dictated that the results were limited to voxel height of *p* < 0.01, cluster-size ≥ 1608 contiguous voxels in accordance with the recent criticism on appropriate statistical threshold [59].

All reported coordinates are in MNI space. Statistical images were overlaid onto the MRIcron (http://www.cabiatl.com/mricro) template image for 3D viewing and xjView (http://www.alivelearn.net/xjview). For ROI based regression analysis and scatter-plots, mean average of cluster that was significant were extracted using the Marsbar toolbox for Matlab (http://web.mit.edu/swg/software.htm).

### Experiment-2: Investigation of chicken or egg in a longitudinal study of beginning readers

#### Participants

Forty-three kindergarten children with varying preliteracy skills and family history of reading difficulty participated in this longitudinal study. Their average age was 5.50 ± 0.31 years-old at the time of the MRI. Their reading skills were assessed again three school years later (Time 2; **Table 2**). All the participants were native English speakers, 26 of them were males, and 39 were right-handed. Ethnicity of families was 5 Asian American. 1 African American, 33 Caucasian American, 1 Hispanic, and 3 Other. Given the study location (Palo Alto, CA), family socioeconomic status was weighted toward high SES, as indexed by income-to-needs ratio (*M* = 8.37, 1.43–33.29), parental education level (*M* = 16.89, 12–22) and occupational status (*M* = 6.48, 2–9), according to 9-point Hollingshead Index Occupational Status Scale [60]. The participants were a subset of a larger group used in our recent studies [61–64]. Among the original sample of 51 children, 8 children were excluded for the following reasons: Three participants were excluded because of movement during their scans. Four participants were lost to follow-up, and one participant had already finished first grade at the time of the scan. Of the 43 participants, 21 met the criteria for a family history of reading difficulty based on the Adult Reading History Questionnaire (ARHQ, [65]), using a cutoff score of greater than 0.4 (for at least one parent), as specified in previous papers [61, 62]. However, at Time 2 (which corresponded to the end of 2^nd^ grade), none of the children were diagnosed with dyslexia and all of them had scores above the 25th percentile rank for both decoding and reading comprehension. Demographic, cognitive, language and reading performance is presented in **Table 2**. None of the children in this study had any parental report of a formal diagnosis of neurological or psychiatric disorders besides specific learning disabilities; they were not on medication and had no contraindications to MRI. All the participants had IQ above 85SS (at or above average IQ), as measured by the Brief Intellectual Ability from Woodcock Johnson Cognitive Battery III (WJ-BIA) [53]. The Stanford University and University of California San Francisco (UCSF) Panels on Human Subjects in Medical Research approved the study, and written informed assent and consent was obtained from each child and guardian. For children between 5 and 6 years of age, the children were orally given an assent but did not sign it, following the guidelines of the Stanford and UCSF Panels on Human Subjects in Medical Research.

**Table 2:** Experiment-2: Demographic and behavioral performance at Time 1 and Time 2 and their correlations with the discrepancy index (DiscInd) at Time 2.

	Mean (SD)		*r*_(43)_ / χ _(32)_ between behavioral measures and DiscInd at Time 2	
	Time 1	Time 2	Time 1	Time 2
Age	5.5 y (0.31)	8.19 y (0.36)	0.21	0.20
Duration	2.69 y (0.19)			0.05
Gender	26 M / 17 F			22.46
Handness	39 R / 4 L			32.01
DiscInd [SS]	-2.51 (14.06)	0.09 (11.12)	-0.05	
DECODE [SS]	109.28 (13.28)	114.81 (13.28)	-0.11	-0.72***
COMP [SS]	106.77 (21.96)	114.91 (9.35)	-0.10	0.16
WJ-BIA [SS]	118.95 (10.67)	116.19 (11.87)	-0.01	-0.05
PPVT [SS]	121.86 (9.67)	119.84 (14.92)	0.16	0.25
WJ-RF [SS]	NA	112.44 (15.92)		-0.19
CTOPP-MD [SS]	104.07 (11.35)	101.86 (13.45)	-0.21	-0.21
TOWRE-PDE [SS]	NA	106.30 (13.76)	NA	-0.40**
TOWRE-SWE [SS]	NA	111.21 (12.12)	NA	-0.23
RAN Average [SS]	104.65 (12.65)^a^	98.84 (12.37)	-0.22	-0.72
WJ-Spelling Fluency [SS]	110.40 (10.45)	105.42 (17.17)	-0.22	-0.34*
TGMV	715.15 (60.69)		0.02	

DiscInd: Discrepancy index, WRMT-PC minus WRMT-WA; DECODE: Woodcock Reading Mastery Test Revised Word Attack Subtest (WRMT-WA); COMP: Woodcock Reading Mastery Test Revised Passage Comprehension Subtest (WRMT-PC); WJ-BIA: Woodcock-Johnson III Cognitive Brief Intelligence Ability Score; PPVT: Peabody Picture Vocabulary Test 3; WJ-RF: Woodcock-Johnson III Reading Fluency; CTOPP-MD: Comprehensive Test of Phonological Processing Memory for Digits Subtest; TOWRE-PDE, Tests of Word Reading Efficiency 2 Phonemic Decoding Efficiency Subtest; TOWRE-SWE: Tests of Word Reading Efficiency 2 Sight Word Efficiency Subtest; RAN Average: Average of Rapid Automatized Naming Color, Object, Letter and Number Subtests; WJ-Spelling: Woodcock-Johnson Spelling Subtest; TGMV: Total Gray Matter Volume; [SS], standard score (Norm = 100, SD = 15); y, years; M, male; F, female; R, right; L, left

*p < 0.05

**p < 0.01

***p < 0.001

NA–not available because norms not available for participants’ age -range

#### Neuropsychological measures

As described in Experiment-1, DiscInd was calculated by subtracting DECODE (as measured by WRMT-WA) from COMP (as measured by WRMT-PC). Additional assessments of language, working memory, reading, and spelling were also administered, as described in Experiment-1. Detailed participant characteristics are provided in Table 2.

#### MRI data acquisition and image processing

**Experiment-2** was also conducted at Stanford University but using a 3 Tesla GE Discovery MR750 scanner with an 8-channel phased array head coil (GE, Milwaukee, WI USA). Three-dimensional high-resolution fast SPGR anatomical scans were acquired: inversion recovery preparation pulse = 400ms, TE = 3.4ms, TR = 8.5ms, FA = 15°, NEX = 1, FOV = 22cm, 128 coronal images, voxel sizes = 0.86 x 1.15 x 1.2mm. For image processing, see **Experiment-1** above.

#### Statistical analyses

In **Experiment-2**, we examined whether brain regions found in **Experiment-1** related to DiscInd is primary or secondary to literacy experience. We therefore performed correlation analyses between Time 1 GMV in beginning readers and their Time 2 DECODE, COMP and DiscInd 3 years later. As in **Experiment-1**, age at time of scan and gender were included as covariates in the model, to control for anatomical differences, as recommended for VBM studies [57]. We used a small-volume correction (SVC) where the ROI was defined as the cluster obtained from **Experiment-1** based on the goal of this study. A statistical threshold of *p* < 0.05 family-wise error (FWE) corrected at the peak level within the ROI was used.

This basic analysis with DiscInd as a dependent variable and age and gender as nuisance variables was further elaborated in three ways. First, nonverbal IQ and reading fluency, which are factors commonly associated with reading outcome, and Time 1 DiscInd and duration between Time 1 and 2, which are confounding factors typically controlled for in longitudinal designs, were used as nuisance variables. Second, some children were able to decode and read sight words at the beginning of kindergarten (Time 1), which may impact their reading outcome 3 years later. Hence, we also controlled for individual differences in the early stages of reading acquisition by including the mean average of word and nonword reading (WRMT Word Identification subtest [WRMT-WID] and WRMT-WA) at Time 1 as a nuisance variable. Third, a regression analysis with DiscInd as the dependent variable was performed, including GROUP (having family history of dyslexia, without family history), GMV of ROI, and GROUP × GMV interaction term as regressors.

### Experiment-3: Domain-general process versus domain-specific language process

In the final set of analyses, we used Neurosynth [40] to attribute function to the results obtained from the experiments above. The Neurosynth database (as of July 2015) contains meta-analytic maps for 3169 terms, automatically derived from coordinates reported in 11,406 papers from selected journals, with a high incidence of functional neuroimaging papers.

We investigated the function of resting state networks associated with the left DLPFC cluster found in **Experiment-1**, using the 1000 Functional Connectome data [66] included in Neurosynth. The map of the average voxel-wise rsFC across 1,000 subjects between the center of gravity (COG) of the left DLPFC region and the rest of the brain was thresholded at a moderate effect size of *r* > 0.30 as in prior studies [67], to limit the analysis to regions that were strongly connected to the seed, and Fisher-transformed. The voxel-by-voxel values from the resulting rsFC map and reverse inference map for each term were correlated. The resulting correlation values are plotted against each key term. In reverse inference analyses, we omitted anatomical terms (e.g. dorsolateral, prefrontal) and averaged terms with common roots or abbreviated forms (e.g., *‘working memory’ and memory wm’*). For each term and voxel within a 2mm resolution standard MNI space, reverse inference maps were constructed by calculating the posterior probability of a term occurring in the text of a study given reported coordinates within 10mm of the voxel, using a uniform prior on term occurrences.

## Results

### Experiment-1: Individual differences in the neural correlates of reading discrepancy in school-aged children

**Fig 1:**
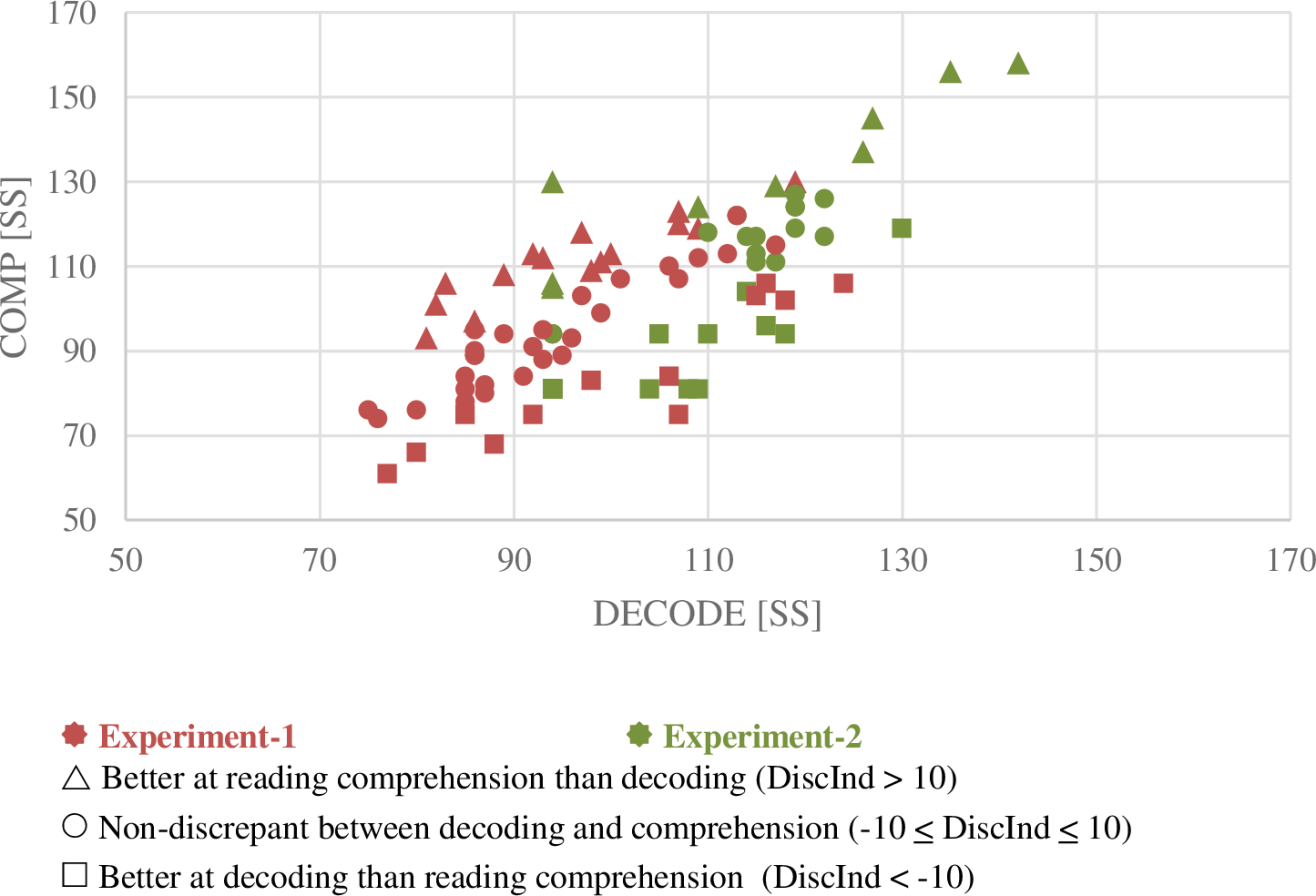
Scatter-plot of decoding (DECODE; WRMT-WA SS) and reading comprehension scores (COMP; WRMT-PC SS). Participants from **Experiment-1** are displayed in **red**. DECODE and COMP measures showed a significant positive correlation (r = 0.67, p < 0.001). The discrepancy index (DiscInd; i.e., COMP “minus (-)” DECODE) was greater than 10 SS (Standard Score) for 27.3% of the participants (**triangles**) i.e., they were better at comprehending than decoding. Discrepancy was in the opposite direction and negative for 21.8% of the participants, i.e., less than -10 SS and they were better at decoding than comprehending (**squares**). Participants that did not belong to either group are those without a large discrepancy between DECODE and COMP (50.9% of the participants, **circles**). Participants from **Experiment-2** at Time 2 are displayed in **green**. DiscInd was greater than 10 SS for 20.9% (**triangles**), less than -10 SS for 41.9% (**squares**), and non-discrepant (between -10 and 10 SS) for 37.2% of the participants (**circles**).

Demographic and behavioral data as well as correlations between measures are included in **Table 1**. DECODE was highly correlated with COMP (*r* = 0.67, *p* < 0.001; **Fig 1, red markers; Table 1**), similar to the correlations found in a behavioral meta-analysis of 110 studies [1]. The Discrepancy index (DiscInd) ranged between -32 and 23SS and was positively correlated with reading comprehension measures such as COMP (WRMT-PC) and GORT-COMP, but not with decoding as measured by DECODE (WRMT-WA) or TOWRE-PDE. DiscInd was associated with language, reading and cognitive skills such as vocabulary (PPVT), reading fluency (GORT rate), and short-term memory span (CTOPP-MD) (**Table 1**). Out of the 12 children with lower reading comprehension relative to their decoding skills, 5 children (41.6%) had a PPVT score lower than 85SS; for those with better reading comprehension relative to their decoding skills, only one child (out of 15, 6.7%) had a PPVT lower than 85SS. This finding is not surprising based on previous studies that showed approximately half of the S-RCD samples also had language impairment [19–21].

#### Main analyses of interest

**Fig 2:**
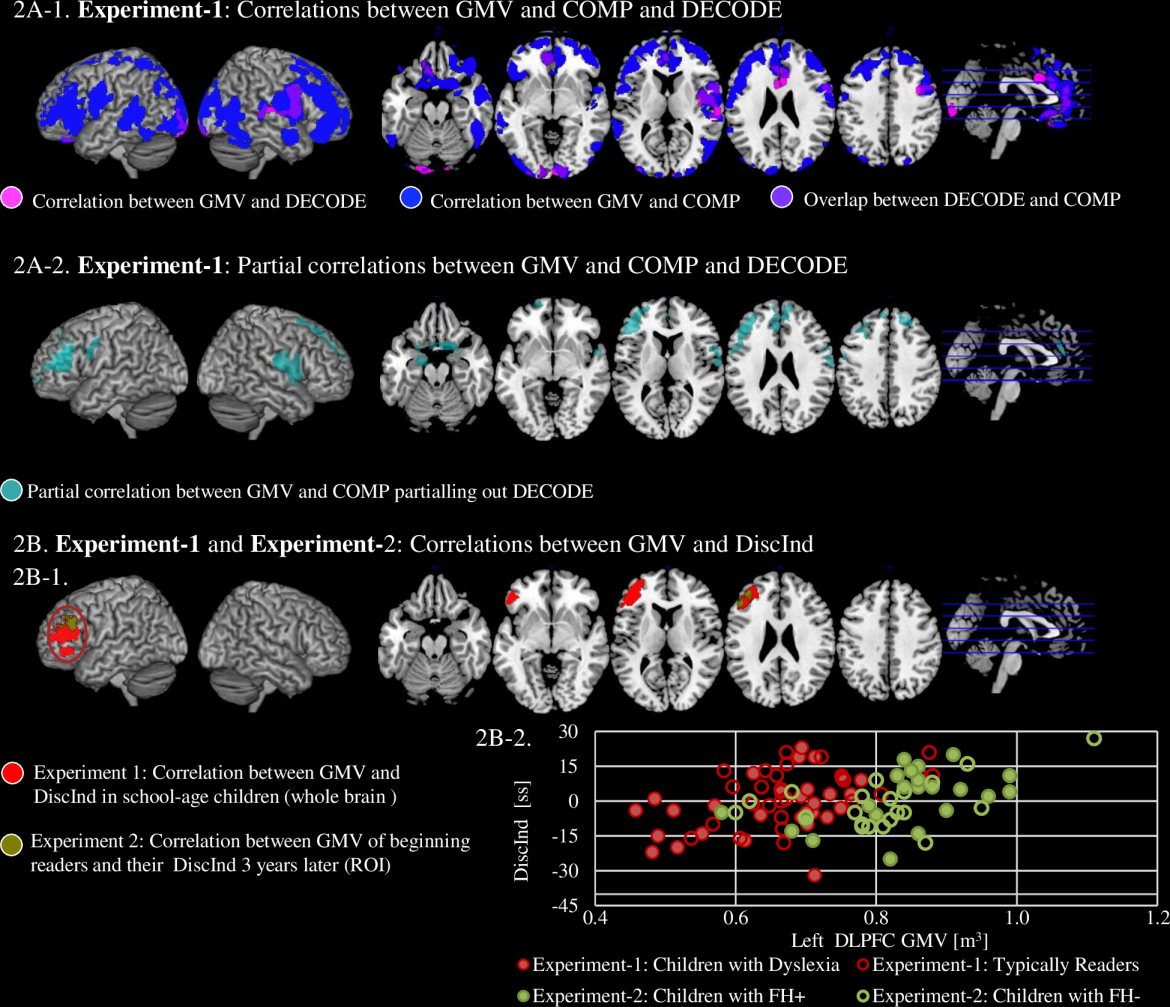
The neural correlates of decoding, reading comprehension and DiscInd. **A. Experiment-1**. **Association between GMV and behavioral measures of decoding (DECODE) and reading comprehension scores (COMP). A-1.** Association between GMV with COMP (blue) and DECODE (pink). **A-2.** Association between GMV with COMP partialling out DECODE (cyan). There were no regions that correlated between GMV and DECODE when COMP was partialled out. **B. Experiment-1 and Experiment-2. Association between GMV and DiscInd, i.e., a score subtracting DECODE from COMP. B-1.** The cluster that shows association between GMV and DiscInd in school-age children from **Experiment-1** whole brain analysis is shown in red. In **Experiment-2,** the cluster that shows association between GMV of beginning readers and DiscInd 3 years later within the cluster obtained from Experiement-1 using ROI small volume correction is shown in green. **B-2.** Mean values extracted from the clusters in **B-1** are plotted as scatterplots to show that the positive relationships between DiscInd scores and left dorsolateral prefrontal cortex (DLPFC) GMV are not driven by children with dyslexia in **Experiment-1** (red full circle) or from the children with a family history of dyslexia in **Experiment-2** (green full circle).

We examined brain regions that correlated with DECODE and COMP. A Whole brain analysis of voxel-by-voxel GMV yielded a significant positive correlation with both DECODE and COMP that extended over large portions of reading-related regions consistent with previous studies, including right inferior frontal gyrus orbitalis (IFGorb), bilateral anterior and middle cingulum, left inferior and middle occipital gyri and bilateral lingual gyri. In addition, only COMP abilities were associated with regions in the bilateral middle frontal gyri (MFG), bilateral inferior frontal gyri pars triangularis (IFGtri), right superior frontal gyrus, bilateral superior, middle and inferior temporal gyri, left superior parietal lobule, and right cerebellum (**Fig 2A-1**, *p* < 0.05 corrected). Of note, a separate whole brain analysis examining DiscInd showed that DiscInd (i.e., greater COMP relative to DECODE) was positively associated with GMV predominantly in the left DLPFC. This cluster included left MFG and IFGtri with the peak centered around MFG (peak MNI coordinates: *x* = -36, *y* = 54, *z* = 15, *Z* = 3.57, *p* < 0.05 corrected, cluster size = 3154 voxels) (**Fig 2B-1, Table 3**).

**Table 3:** Experiment-1: Brain regions where gray matter was associated with the discrepancy index (DiscInd).

Brain region	Brodmann area	MNI coordinates			*t* values (peak)	Cluster size (voxels)
		*x*	*y*	*Z*		
Positive correlation with DiscInd						
Left Middle frontal gyrus, inferior frontal gyrus pars triangularis	46, 10	-36	54	15	3.57	3154
		-51	32	19	3.64	
		-45	44	10	3.25	

#### Control analyses

Four control analyses were performed. First, correlation in the left DLPFC GMV remained significant after partialling out DECODE from COMP (peak MNI coordinates: *x* = -42, *y* = 33, *z* = 36, *Z* = 4.08, *p* < 0.05 corrected, voxel size = 2380 voxels). Other regions such as bilateral IFGtri, and IFGorb right superior frontal gyrus, right anterior cingulum and bilateral superior medial frontal gyrus were also associated with COMP after partialling out DECODE (**Fig 2A-2**). However, as expected, no cluster remained when COMP was partialled out from DECODE. Thus, the left DLPFC GMV was associated with reading comprehension above and beyond decoding, and with reading comprehension-decoding discrepancy. Second, the discrepancy-related region remained significant when DiscInd-GORT was a dependent variable (peak MNI coordinates: *x* = -38, *y* = 51, *z* = 13, *Z* = 3.57, *p* < 0.05 corrected, cluster size = 3761 voxels). Third, a partial correlation between the left DLPFC cluster and DiscInd remained significant, even after we regressed out variables that were associated with reading comprehension, such as nonverbal IQ and reading fluency (*β* = 0.35, *p* = 0.007). Note that age and gender were controlled in all analyses. Fourth, as can be seen in the scatterplots of the left DLPFC, DiscInd and group relationships (illustrated in **Fig 2B-2, red**), DiscInd was only correlated with GMV in the left DLPFC (*β* = 0.42, *t* = 2.10, *p* = 0.04) and was not correlated with Group (*β* = -0.06, *t* = -0.46, *p* = 0.65) or GMV by Group interaction (*β* = 0.035, *t* = 0.18, *p* = 0.86).

### Experiment-2: Investigation of chicken or egg in a longitudinal study of beginning readers

To address whether greater left DLPFC GMV found in **Experiment-1** predates reading acquisition and is more causally related to discrepancy, or is due to secondary consequences or literacy experience, we examined whether left DLPFC GMV in beginning kindergarteners can predict reading discrepancy 3 years later. Behaviorally, as in the other dataset, DECODE was significantly correlated with COMP (Time 1, *r* = 0.79, *p* < 0.001; Time 2, *r =* 0.67, *p* < 0.001, see **Fig 1, green; Table 2**). Unlike **Experiment-1** where DiscInd correlated positively with COMP but not with DECODE, in this sample, DiscInd was not associated with DECODE or with COMP. Time 2 DiscInd was negatively correlated with Time 2 DECODE and with another measure of nonword reading (TOWRE-PDE) (**Table 2**). However, neither Time 1 DiscInd nor other Time 1 behavioral measures predicted Time 2 DiscInd.

ROI analysis within the DLPFC cluster derived from **Experiment-1** examining the effect of DiscInd indicated that Time 1 left DLPFC GMV in beginning readers was positively associated with Time 2 DiscInd (peak left MFG MNI *x* = -50, *y* = 30, *z* = 27, *Z* = 3.57, *p* < 0.05 corrected, 416 voxels) (**Fig 2B-1, green**). As expected, Time 1 left DLPFC GMV did not correlate with DECODE or COMP. Moreover, positive correlation in the left DLPFC remained significant even after partialling out key factors (IQ, reading fluency and DiscInd at Time 1 as well as duration between Time 1 and 2) (peak left MFG MNI *x* = -50, *y* = 30, *z* = 27, *Z* = 3.32, *p* < 0.05 corrected, 137 voxels) or controlling for individual difference in reading skills at the early stages of reading acquisition (peak left MFG MNI *x* = -50, *y* = 30, *z* = 27, *Z* = 3.87, *p* < 0.05 corrected, 456 voxels).

Further, we tested whether family history of reading difficulties interacted with reading discrepancy in regional GMV. Mean GMV in left DLPFC derived from the basic analysis of **Experiment-2** was only correlated with DiscInd (*β* = 0.55, *t* = 2.51, *p* = 0.017) and was not correlated with Family History (*β* = 0.09, *t* = -0.627, *p* = 0.53) or the DiscInd x Family History interaction (*β* = 0.035, *t* = 0.16, *p* = 0.86) (**Fig 2B-2, green**).

### Experiment-3: Domain-general versus domain-specific language processes

**Fig 3:**
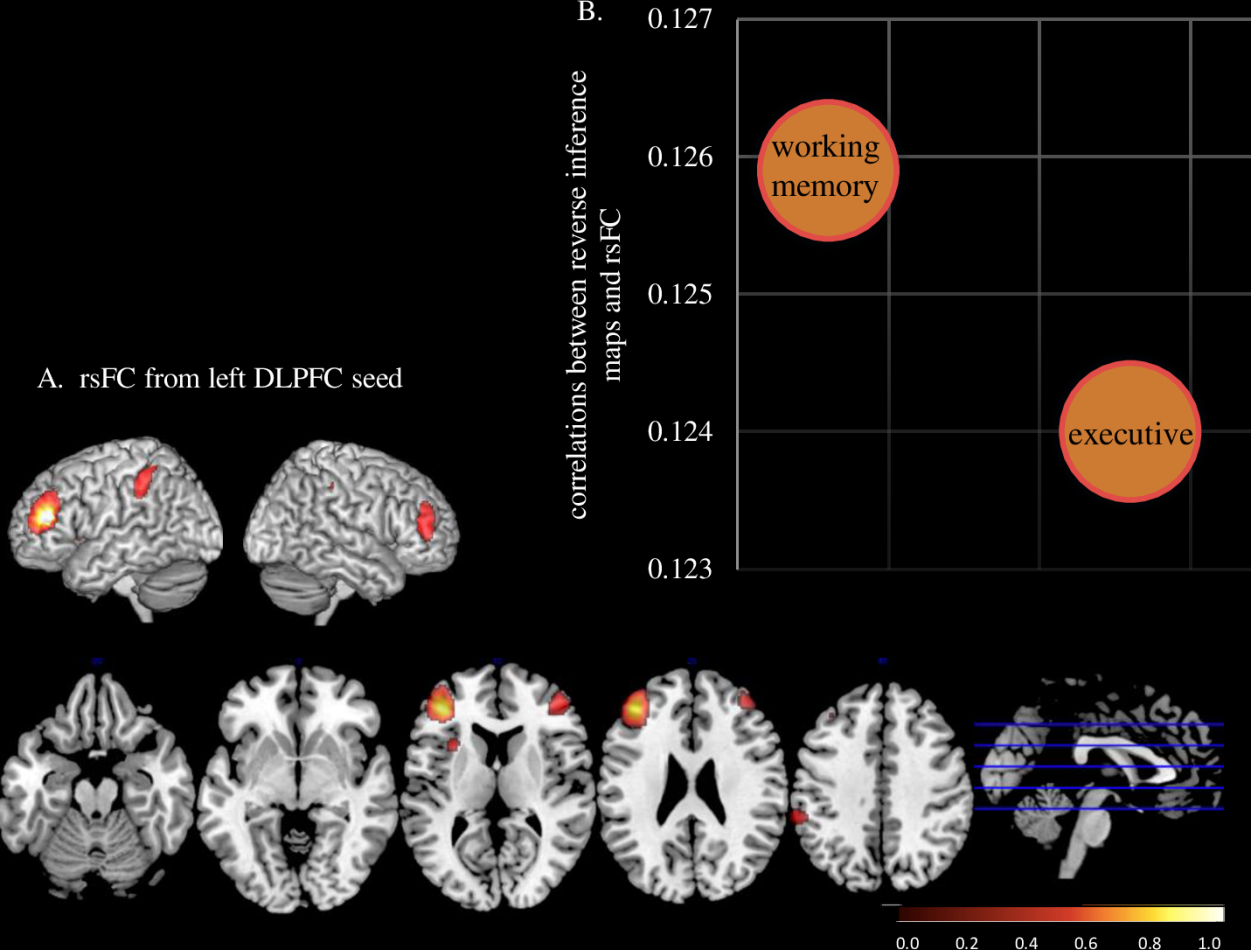
Reverse inference results in Experiment-3. **A.** Resting state functional connectivity (rsFC) from the left DLPFC cluster’s center of gravity (COG) as seed (MNI x = -40, y = 38, z = 16) using the 1000 Functional Connectome data using threshold of r > 0.30 **B.** The voxel-by-voxel values from the resulting rsFC map and reverse inference map for each term were correlated. The resulting correlation values are plotted against each key term with threshold of r > 0.1.

Using the resting state fMRI data from the 1000 Functional Connectome Project [66] with the aforementioned left DLPFC identified in **Experiment-1** (COG of cluster: MNI *x* = -40, *y* = 38, *z* = 16) as a seed voxel revealed a bilateral (left > right) fronto-parietal network including superior frontal gyri, insula, and supramarginal gyri [66] (**Fig 3A**). Reverse inference of the rsFC network associated with the DLPFC cluster identified two terms that related to cognitive control (‘executive’) or working memory (‘working memory’) (**Fig 3B**).

## Discussion

A large number of studies have investigated the neural correlates of developmental reading disorders. Nevertheless, only few studies examined the neural basis of readers that show a discrepancy between two important reading skills: decoding and reading comprehension. While these skills are highly related, the existence of discrepant readers suggests that there are additional skills important to becoming proficient at reading comprehension. Our main goal was to fill this gap and examine the neural basis that underlie the discrepancy between decoding skills and reading comprehension in children with a wide range of reading abilities.

In all the experiments, we found converging evidence of the left DLPFC to be critical in the development of having discrepantly higher reading comprehension relative to their decoding ability. First, in school-age children with a wide range of reading abilities, the left DLPFC GMV was associated with increased discrepancy between reading comprehension and decoding above and beyond contributions from decoding, reading comprehension measures, diagnosis of dyslexia and other related reading and cognitive skills (**Experiment-1)**. This association with the left DLPFC was similar across the entire continuum of reading discrepancy, with those having low decoding skills but relatively good comprehension skills at one end and those with good decoding skills but poor comprehension skills at the other end. When confounding variables (nonverbal IQ and reading fluency) were controlled, this relationship remained significant. Moreover, in a small and preliminary study reported in the **S1 File**, resilient dyslexics (i.e, those with poor decoding but with, unexpectedly, good reading comprehension) had greater left DLPFC GMV compared to both groups of children matched for poor decoding (i.e., those with poor, but no discrepancy) and those matched for good reading comprehension (i.e., those with good decoding was also good, also without a discrepancy), providing further evidence that the left DLPFC uniquely contributes to this discrepancy. Second and most intriguingly, using a longitudinal design in beginning readers (5–6 year old), a region within the left DLPFC that was identified in **Experiment-1** predicted reading discrepancy 3 years later, above and beyond reading skills at the beginning stages of reading (**Experiment-2**). When confounding variables (IQ, reading fluency, DiscInd at Time 1 as well as duration between Time 1 and 2) were controlled, this relationship remained significant. Moreover, the findings showing that reading group (dyslexia or family history) did not interact with GMV, as well as the fact that all the participants in **Experiment-2** had within or above normal decoding skills, support that the left DLPFC is not specific to dyslexic readers, but also to individuals across a range of decoding skills. Finally, the seed voxel connectivity analysis using the 1000 Functional Connectome data identified the left DLPFC node to be part of the fronto-parietal network (**Experiment-3**). Within a meta-analytic reverse inference framework, the fronto-parietal network derived from rsFC was related only to domain-general processes. Taken together, these findings indicate that a left DLPFC network critical for memory and cognitive control may play a key role in the development of reading comprehension regardless of decoding, even before a child becomes a proficient reader.

It is currently unclear how the left DLPFC mediates the discrepancy between decoding skills and reading comprehension abilities. The DLPFC network has been found to be involved in decoding, dyslexia [68, 69], reading comprehension [70, 71], and in the decoding—reading comprehension discrepancy, when reading comprehension is discrepantly high (resilient dyslexics) [34] to low (S-RCD) [17, 35]. However, these studies did not address questions such as the brain regions that are unique to the comprehension-decoding discrepancy, the role of these brain regions, specifically whether DLPFC accounts for the development of good comprehension despite poor decoding, and whether the involvement of DLPFC is associated with long-term reading experience (or lack of it thereof) and a secondary consequence of the enhanced use of compensatory strategies. A possible functional role of this brain region was identified through **Experiment-3**, where fronto-parietal networks, identified using rsFC, were associated with working memory and cognitive control. Namely, these cognitive processes may be key constructs enabling children to be proficient in higher level reading processes (comprehension) despite their relatively poor abilities in lower level reading processes (decoding). This finding corroborates with the significant behavioral correlation of DiscInd and the verbal short-term memory span (**Experiment-1**) which reflects that the ability to temporarily hold verbal information and is considered to be a subsystem to the working memory [72, 73] Moreover, previous behavioral studies indicate that working memory and cognitive control play crucial roles in moderating reading comprehension and decoding relationships [24, 48]. For example, planning abilities, attention and working memory, have shown to support reading comprehension directly above and beyond foundational reading and language abilities [28]. Furthermore, children with S-RCD show prominent weaknesses in executive function [26]. Intervention studies report that training working memory enhances reading abilities [74], although it is controversial [75]. **Experiment-3** supports the notion that language skills alone cannot account for the comprehension-decoding discrepancy and suggests the importance of further research to examine the role of cognitive skills in this discrepancy. These findings are in line with a recently proposed theoretical framework, suggesting that a core set of 'language' regions may interact functionally with a set of domain-general regions during the performance of language tasks [76]. In particular, left DLPFC may provide top-down and predictive strategies for language comprehension [77].

Our longitudinal findings show that, as children begin formal reading instruction in kindergarten, individual differences in left DLPFC GMV are predictive of who will become better reading comprehenders above and beyond their decoding ability, at an age when they are expected to be proficient readers (**Experiment-2**). Together with the preliminary findings of the **S1 File** that show uniquely greater left DLPFC GMV in resilient dyslexics compared to two control group**s**, it is possible that differences in DLPFC at beginning stages of reading enable buffering of superior reading comprehension abilities relative to their level of decoding, and even when the decoding skills are poor.

Our study contributes to the brain and cognitive reserve framework, as well as to our recent proposal for a framework of cognitive (and socio-emotional) resilience in dyslexia [78]. Reserve has been proposed mostly in the aging literature as a cognitive and/or neural mechanism that allows some to cope better than others despite similar brain deficits [79]. Our recent proposal of cognitive resilience in dyslexia has been suggested that several cognitive processes act as protective factors that reduce the severity of dyslexia [78]. Of relevance, executive functions were found to be key protective factors [80–82]. Our finding that the left DLPFC GMV boosts reading comprehension above and beyond decoding is in alignment with previous studies in adults that found the left DLPFC network to be a key region important for cognitive resilience [83]. Our study suggests, for the first time, a role of the PFC that may buffer children to outperform in reading comprehension given a certain proficiency in decoding. In sum, we present the first evidence in neurodevelopmental disorders that supports the idea of brain reserve [84] and first imaging evidence of the neural correlates of cognitive resilience in dyslexia.

**Fig 4:**
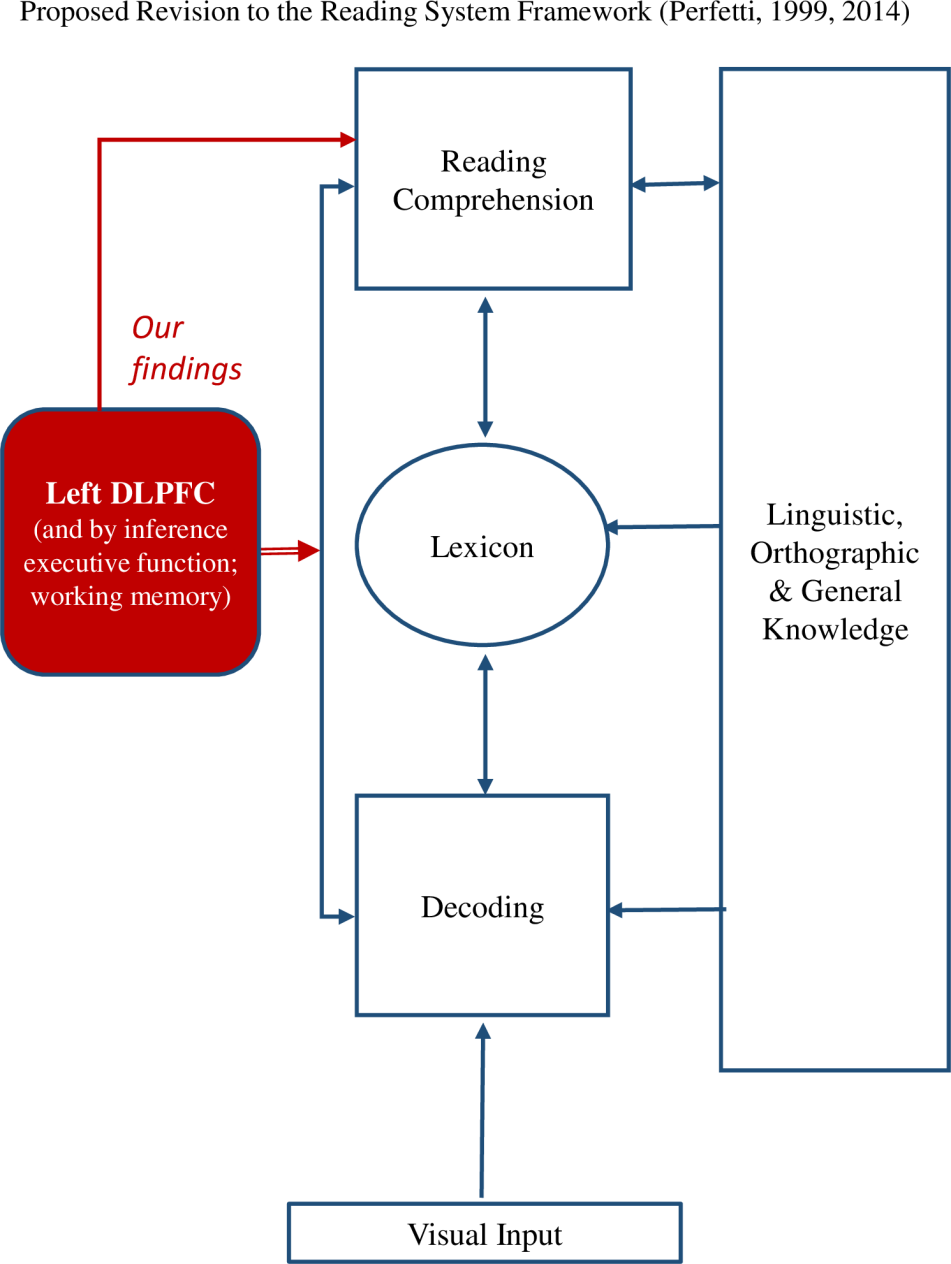
Description of the Reading System Framework and contributions of the current study to this Framework. The **Reading System Framework** by Perfetti is represented in **blue lines.** It emphasizes the lexical component that mediates decoding, reading comprehension and the integration between language knowledge with reading processes. **Current findings** are represented in **red lines.** They indicate that the left DLPFC network, and possibly by inference, cognitive control and working memory, influence reading comprehension (**single-lined red arrow**), also strongly modify the strength of the relation between the two (**double-lined red arrow**).

Of importance, understanding the neural systems underlying individual differences in the reading comprehension-decoding relationship may refine theoretical models of reading. Expanding the Simple View of Reading [15, 16], Cutting et al. [85] suggests that executive function facilitates the growth and intertwining of decoding and language abilities to achieve skilled reading comprehension [25, 85]. Further, Perfetti’s reading system framework [86, 87] proposes that the lexicon mediates the relationship between decoding and reading comprehension, and that reading requires integration between language knowledge (e.g., letter knowledge, facts about the world) with reading processes (e.g., decoding, comprehension monitoring; see in **Fig 4, blue**). A complementary model, the Construction-Integration Model by Kintsch and colleagues, proposed that working memory plays a critical role in this integration [30]. In the context of these models, findings from the current study supports the idea that the left DLPFC is associated with discrepancy for the entire spectrum of decoding ability (**Experiment-1**). Relevant to these models, our findings also indicate that the left DLPFC is associated with discrepancy rather than decoding or reading comprehension on their own (**Experiment-1**). Resilient dyslexics had greater left DLPFC even compared to good comprehenders who had similar (and above) reading comprehension abilities along with good decoding skills (**S1 File**), and the left DLPFC region was associated only with discrepancy and not with reading comprehension or decoding 3 years later (**Experiment-2**). Together with results from the reverse inference linking the left fronto-parietal network to cognitive control and working memory (**Experiment-3**), our findings may imply that domain-general processes influence reading comprehension (**Fig 4, single-lined red arrow**), but also strongly modify the strength of the relation between decoding and reading comprehension (**Fig 4, double-lined red arrow**). It is possible that greater cognitive resources serve to boost reading comprehension, given a limited level of decoding. We did not find the DLPFC to be involved in decoding with the current threshold, (but it is involved when a smaller smoothing kernel is used), which is in line with previous studies that also showed the involvement of the DLPFC network in reading comprehension but not decoding (or only weak relationships). [3, 33, 85]

While the current study is the first comprehensive report on the neurobiological substrates of reading discrepancy, it has several limitations. First, a major limitation of this study is the few behavioral measures on working memory that could have been used to directly test the process underlying reading discrepancy. We believe that we have minimized this issue by using an approach that combines 1000 Functional Connectome data and large-scale reverse inference using Neurosynth. Future investigations should include measures such as language, executive functions, and task-driven functional neural circuits. Second, since our overall sample sizes were relatively small, we could not conduct between-group analyses comparing for example, unique profiles of reading disorders such as resilient dyslexics and S-RCD (but see **S1 File**). Further investigations, including larger groups of resilient dyslexics, poor comprehenders, and non-discrepant good and poor readers, are warranted. Third, we used a liberal primary threshold of *p* < 0.01 uncorrected for height when computing a corrected threshold at *p* = 0.05, which could be a source of inflated false positive rates [59]. Our use of a large smoothing kernel (resulting in a smoothness of approximately 16mm FWHM) minimizes this issue by imposing a Gaussian spatial correlation structure (and stationarity). This is a larger degree of smoothing than typically employed in 2mm^3^ fMRI data. Although it is possible that VBM smoothness is not approximated by a Gaussian, we are unaware of any studies of the smoothness distribution in VBM data, particularly the presence of the long-tailed distribution found in fMRI data that is particularly problematic for cluster-based thresholding. Further studies with larger groups of children would help to produce these analyses with a more stringent statistical threshold. Fourth, temporal precedence in beginning readers (**Experiment-2)** does not imply causation, and future examination is warranted prior to attribution of causality. Fifth, we calculated the main dependent measure (DiscInd) by the difference between DECODE and COMP to enhance generalizability of our findings (see **Experiment-1 Methods** for details). We nevertheless performed the alternative analysis as well, calculating residuals of COMP partialling out DECODE (**Fig 2A-2**), and found similar results. Finally, we would ultimately need updated computational models and formal theories to explain our findings.

In summary, our comprehensive neuroimaging examination of reading-discrepancy using good to poor and beginning to advanced readers, with cross-sectional and longitudinal data, converged on the left DLPFC as one key anatomical region that buffered children from having poor reading comprehension skills that might be expected based on their decoding ability. In other words, a domain-general network including a left DLPFC node appears to play a protective role that aids reading comprehension in the face of poor(er) decoding abilities. Our findings also demonstrate the prognostic capabilities of brain imaging by showing how left DLPFC volume in beginning readers was associated with discrepancy three years later. Collectively, our study provides insight into reading development, and refines existing theories of reading. Also, with future work in larger samples of resilient dyslexics and replication as well as prospective intervention studies, the current study can potentially inform the practice of reading disorders, especially as it relates to thinking about strategies to improve the downstream negative consequences of decoding difficulties.

## Supporting information

S1 File Brain basis of cognitive resilience: Prefrontal cortex predicts better reading comprehension in relation to decoding. (DOCX)
