# Supplementary material for: Brain basis of cognitive resilience: Prefrontal cortex predicts better reading comprehension in relation to decoding
Source: PLoS One. 2018 Jun 14;13(6):e0198791. doi: 10.1371/journal.pone.0198791 (PMC6002103; doi:10.1371/journal.pone.0198791)
Supplement: S1 File — (DOCX) [file pone.0198791.s001.docx]

**Brain basis of cognitive resilience: Prefrontal cortex predicts better reading comprehension in relation to decoding – Supporting Appendix**

Smadar Z. Patael^1,2^, Emily A. Farris^1,3^, Jessica M. Black^4^, Roeland Hancock^1^, John D. E. Gabrieli^5,6^, Laurie E. Cutting^7,8^, Fumiko Hoeft MD^1,8,9*^

Neural specify of reading discrepancy

To fully disambiguate the effect of reading comprehension and discrepancy between reading comprehension and decoding that correlate highly in our sample, we performed supplementary but critical analyses with a subsample of children from **Experiment-1** (n = 36 of 55), despite the sample-size being small. We compared gray matter volume (GMV) between three groups: 1) Resilient dyslexics (ResilientD) who have low decoding and average reading comprehension skills with a discrepancy between the two, 2) Poor Readers (PoorR) who have low decoding and reading comprehension skills without a discrepancy, and 3) Good Readers (GoodR) who have average decoding and reading comprehension skills, also without discrepancy. This analysis provided a preliminary but crucial complement to the analyses provided in the main text. First, the analysis provides additional support for the neural correlates of discrepancy between reading comprehension and decoding. In addition, by including the GoodR group matched on reading comprehension to ResilientD, it provides clear evidence that any findings found here are not related to ResilientD having better reading comprehension (than the PoorR group). Conversely, by inclusion of the PoorR group matched on decoding to ResilientD, it provides clear evidence that any findings found here are not related to ResilientD having poorer decoding (than the GoodR group). Thus, the inclusion of the two control groups allowed the disambiguation of the finding that it is indeed related to discrepancy, and not to good comprehension or poor decoding.

***Participants & group criteria:*** Children were placed into one of three groups (ResilientD, PoorR and GoodR) based on composite scores of decoding skills and reading comprehension as it has been recommended to identify poor skills ([1](#_ENREF_1)): 1) DECODE’: an average composite score on nonword reading (from Woodcock Reading Mastery Test Revised Word Attack Subtest [WRMT-WA] and Tests of Word Reading Efﬁciency Phonemic Decoding Efﬁciency Subtest [TOWRE-PDE]) ([2](#_ENREF_2), [3](#_ENREF_3)). 2) COMP’: an average composite score on reading comprehension (based on Woodcock Reading Mastery Test Revised Passage Comprehension Subtest [WRMT-PC] and Gray Oral Reading Tests 3 Comprehension Subtest [GORT-COMP]). ([4](#_ENREF_4)) 3) DiscInd’: Discrepancy between nonword reading composite and reading comprehension composite, and 4) Formal diagnosis of dyslexia for ResilientD and PoorR.

ResilientD (n = 8) with a formal diagnosis of dyslexia had nonword composite below 87 Standard Score [SS] , reading comprehension composite of 95SS and above, and individual DiscInd’ greater than SS of 10. PoorR (n = 13) with a formal diagnosis of dyslexia were matched for nonword reading to the ResilientD group; they had a nonword reading composite below 87 SS, reading comprehension below 90 SS, and DiscInd’ less than 10 SS. GoodR (n = 15) with no history of dyslexia were matched for reading comprehension scores in the ResilientD group; they had a nonword reading score above 92 SS, COMP’ 95 SS and above, and DiscInd’ less than 10 SS. While the sample was small for ResilientD, this analysis disambiguates the effect of DiscInd’ from COMP’ and allowed us to examine brain regions that respond specifically to DiscInd’. Following these criteria, 36 out of 55 participants in **Experiment-1** were included in this analysis. The reasons for excluding 19 participants of the group analysis were as follows. Eight participants did not match ResilientD or GoodR groups, since they had good decoding skills and DiscInd greater than 10SS. Five participants fell in an intermediate range of 87 - 92 SS, so we did not consider them as having good or poor decoding skills. Two participants had an S-RCD profile. To eliminate alternative explanation, that is, that ResilientD had better decoding skills than PoorR, we excluded the three worst decoders in the PoorR group, thus matching decoding performance in both groups. Additionally, since our total sample was quite small, and in particular ResilientD (n = 8), we tried to avoid including outliers in the control groups. Therefore, we excluded one participant from the GoodR group for having extremely good reading comprehension skills of a SS of 127.5. **Fig S1** presents the decoding composite and reading comprehension scores of the entire sample included in **Experiment-1,** together with the participants that were excluded from the group analyses in this **Supporting Appendix.**

***Statistical analyses:*** A one-way analysis of variance compared GMV between ResilientD, PoorR and GoodR within a region of interest (ROI), which was defined as the left dorsolateral prefrontal cortex (DLPFC) cluster obtained in **Experiment-1**. A Small-volume correction (SVC) was applied using a statistical threshold of *p* < 0.05 Family-Wise Error (FWE) at the peak level.

***Results and Discussion*:** Age, gender and handedness were not reliably associated with group membership (all *p's* > 0.05). For reading measures such as reading fluency, language and cognitive measures, one-way ANOVAs for language and other cognitive measures showed significant main effects of group (all *p’s* < 0.01), primarily driven by expected differences based on how groups were defined, and consistent with past literature on reading disorders (**Fig S2 A through C**, **Table S1**). Compared to PoorR, ResilientD also exhibited significantly better sight word reading and receptive vocabulary measured by TOWRE Sight Word Efﬁciency Subtest (TOWRE-SWE) and the Peabody Picture Vocabulary Test, 3 (PPVT) respectively ([3](#_ENREF_3), [5](#_ENREF_5)), which is consistent with past literature on resilient dyslexics ([6](#_ENREF_6)). Intriguingly, ROI analysis of the significant clusters from **Experiment-1** showed that the left DLPFC GMV was increased in ResilientD compared to *both* PoorR and GoodR (left middle frontal gyrus: peak MNI *x* = -28, *y* = 35, *z* = 21, *Z* = 3.43, *p* < 0.05 corrected, cluster size = 11 voxels; **Fig S2-D**). This was the case despite the fact that PoorR were matched on DECODE’, and GoodR were matched on COMP’ to ResilientD, allowing us to conclude that greater left DLPFC GMV is associated with reading discrepancy above and beyond reading comprehension and decoding.

GMV

**Table S1. Demographic and behavioral performance of resilient dyslexics (ResilientD), poor readers (PoorR) and good readers (GoodR)**

|  | ResilientD (***n***=8) | PoorR  (***n***=13) | GoodR (***n***=15) | ***F***_(2,35)_/ χ_(2)_ | ResilientD vs PoorR^a^ | ResilientDvs GoodR^a^ | PoorR vs GoodR^a^ |
| --- | --- | --- | --- | --- | --- | --- | --- |
| Age | 14.76 (1.34) | 14.69 (1.08) | 13.98 (2.53) | 0.68 | ns | ns | ns |
| Gender | 5 M | 10 M | 8 M | 1.69 |  |  |  |
| Handedness | 7 R | 13 R | 15 R | 1.54 |  |  |  |
| DiscInd’ | 19.31 (7.57) | 1.50 (5.52) | 1.00 (7.06) | 23.04*** | *** | *** | ns |
| DECODE’ [SS] | 83.19 (2.67) | 80.42 (3.99) | 108.67 (8.16) | 91.43*** | ns | *** | *** |
| COMP’ [SS] | 102.50 (6.47) | 81.92 (6.54) | 109.67 (6.6) | 64.82*** | *** | ns | *** |
| WASI-MR [SS] | 105.63 (11.78) | 100.77 (12.56) | 116.33 (6.67) | 8.34** | ns | ns | ** |
| PPVT [SS] | 102.75 (10.62) | 83.15 (12.62) | 110.07 (15.41) | 14.268*** | ** | ns | *** |
| CTOPP-MD [SS] | 101.25 (15.53) | 88.85 (13.1) | 113.93 (13.47) | 11.12*** | ns | ns | *** |
| WRMT-WID [SS] | 85.25 (2.87) | 77.62 (6.09) | 111.20 (13.32) | 47.08*** | ns | *** | *** |
| WRMT-WA [SS] | 86.38 (3.42) | 86.00 (4.3) | 109.53 (8.01) | 65.75*** | ns | *** | *** |
| WRMT-PC [SS] | 97.50 (7.09) | 80.77 (8.47) | 109.33 (8.48) | 42.33*** | *** | ** | *** |
| GORT Rate [SS] | 88.13 (6.51) | 73.85 (7.68) | 99.33 (18.01) | 13.46*** | ns | ns | *** |
| GORT Accuracy [SS] | 76.88 (8.84) | 68.46 (10.08) | 118.93 (13.33) | 75.05*** | ns | *** | *** |
| GORT Fluency [SS] | 73.75 (8.35) | 61.92 (7.51) | 108.00 (33.69) | 15.30*** | ns | ** | *** |
| GORT-COMP [SS] | 107.50 (8.45) | 83.08 (7.78) | 110.00 (8.45) | 42.01*** | *** | ns | *** |
| TOWRE-PDE [SS] | 80.00 (3.34) | 74.85  (5.4) | 107.80 (9.49) | 83.43*** | ns | *** | *** |
| TOWRE-SWE [SS] | 90.38 (5.73) | 79.08 (8.14) | 103.38 (6.5) | 38.91*** | ** | *** | *** |
| RAN Average [SS] | 103.88 (8.59) | 101.62 (10.24) | 114.64 (12.16) | 5.49** | ns | ns | ** |
| WJ-Spelling [SS] | 81.00 (3.21) | 76.62 (10.32) | 109.43 (11.96) | 41.10*** | *** | *** | *** |
| WJ-Writing Fluency[SS] | 103.38 (8.68) | 89.77 (11.2) | 110.14 (8.97) | 14.83*** | * | ns | *** |
| TMGV | 665.83 (49.82) | 704.96 (60.92) | 662.10 (62.9) | 1.90 | ns | ns | ns |

DiscInd’: Discrepancy index, Reading comprehension composite minus decoding composite; DECODE’: Decoding composite, average of WRMT-WA and TOWRE-PDE; COMP’: Reading comprehension composite, average of WRMT PC and GORT Comprehension; WASI-MR: Wechsler Abbreviated Scale of Intelligence Matrix Reasoning Subtest; PPVT: Peabody Picture Vocabulary Test; CTOPP-MD: Comprehensive Test of Phonological Processing Memory for Digits Subtest; WRMT-WID: Woodcock Reading Mastery Test Revised Word Identiﬁcation Subtest; WRMT-WA: Woodcock Reading Mastery Test Revised Word Attack Subtest; WRMT-PC: Woodcock Reading Mastery Test Revised Passage Comprehension Subtest; GORT Rate: Gray Oral Reading Tests 3 Rate Subset; GORT Accuracy: Gray Oral Reading Tests 3 Accuracy Subtest; GORT Comprehension: Gray Oral Reading Tests 3 Comprehension Subtest; TOWRE-PDE: Tests of Word Reading Efﬁciency 2 Phonemic Decoding Efﬁciency Subtest; TOWRE-SWE: Tests of Word Reading Efﬁciency 2 Sight Word Efﬁciency Subtest; RAN Average: Average of Rapid Automatized Naming Color, Object, Letter and Number Subtests; WJ Spelling: Woodcock-Johnson Spelling Subtest; WJ Writing Fluency: Woodcock-Johnson Writing Fluency Subtest; TGMV: Total Gray Matter Volume; [ss] standard score (Norm = 100, SD = 15); y, years; M, male; F, female; R, right; L, left;

**p* < 0.05; ***p* < 0.01; ****p* < 0.001; ns - not significant;

^a^ Bonferroni corrected

**(INSERT FIG S1 AND S2 APPROXIMATELY HERE)**

***Fig S1. Scatterplot showing group selection based on composite decoding (DECODE') and composite reading comprehension (COMP’)***

*The three groups identified here* *are subsamples from* ***Experiment-1****, i.e., a subsample of the Resilient dyslexics (ResilientD) is highlighted in red, Poor Readers (PoorR) in blue, and Good Readers (GoodR) in green. A dash line indicated the criteria we used as the cutoff point for DECODE’ and COMP’. DECODE’: Average of TOWRE–PDE (Phonemic Decoding Efficiency) and the WRMT–WA (Word Attack) SS; COMP’: Mean average of GORT – reading comprehension and WRMT - passage comprehension SS.*

***Fig S2. Behavioral and neuroanatomical patterns that differ between resilient dyslexics (ResilientD), poor (PoorR) and good readers (GoodR)***

***A.*** *DECODE’, nonword decoding composite* ***B.*** *COMP’, reading comprehension composite,* ***C.*** *DiscInd’, discrepancy between the two behavioral measures (COMP’ – DECODE’), and* ***D.*** *Left DLPFC GMV that showed significant differences between groups. Left DLPFC is derived from the main results in* ***Experiment-1*** *(left MFG: peak MNI x = -28, y = 35, z = 21, Z = 3.43, p < 0.05 corrected, cluster size = 11 voxels) that showed significantly greater GMV in ResilientD, compared to PoorR and GoodR. Error bars represent standard error of the mean (SE).*

**References**

1. Keenan JM, Hua AN, Meenan CE, Pennington BF, Willcutt E, Olson RK. Issues in identifying poor comprehenders. Top Cogn Sci. 2014;114:753-77.

2. Woodcock RW. Woodcock diagnostic reading battery: Itasca, IL: Riverside; 1997.

3. Torgesen JK, Wagner R, Rashotte C. TOWRE–2 Test of Word Reading Efficiency. Austin, TX: Pro-Ed; 1999.

4. Weiderholt J, Bryant B. GORT-3: Gray Oral Reading Test. Austin, TX: PRO-ED; 1992.

5. Dunn LM, Dunn LM. PPVT-III: Peabody picture vocabulary test: American Guidance Service Circle Pines, MN; 1997.

6. Welcome SE, Chiarello C, Halderman LK, Leonard CM. Lexical processing skill in college-age resilient readers. Read Writ. 2009;22(3):353-71.

**Fig S1**

**
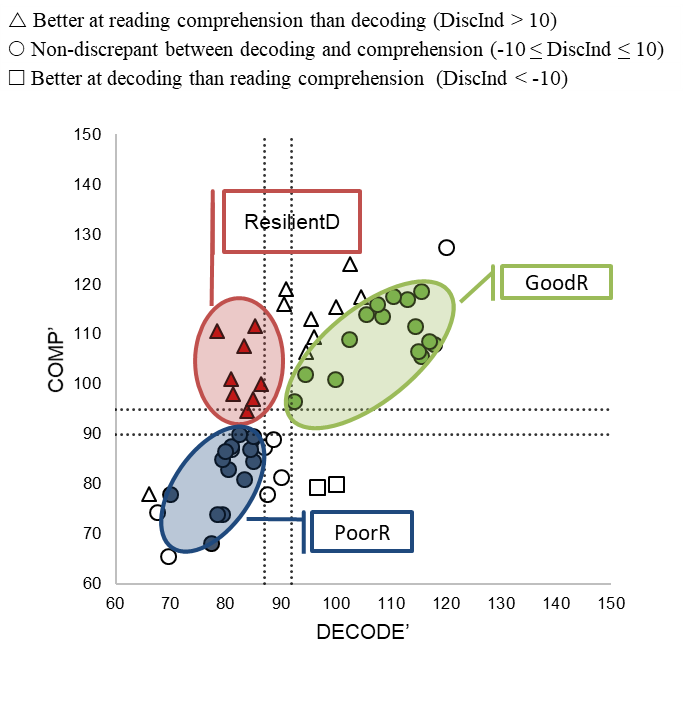
**

**Fig S2**

**
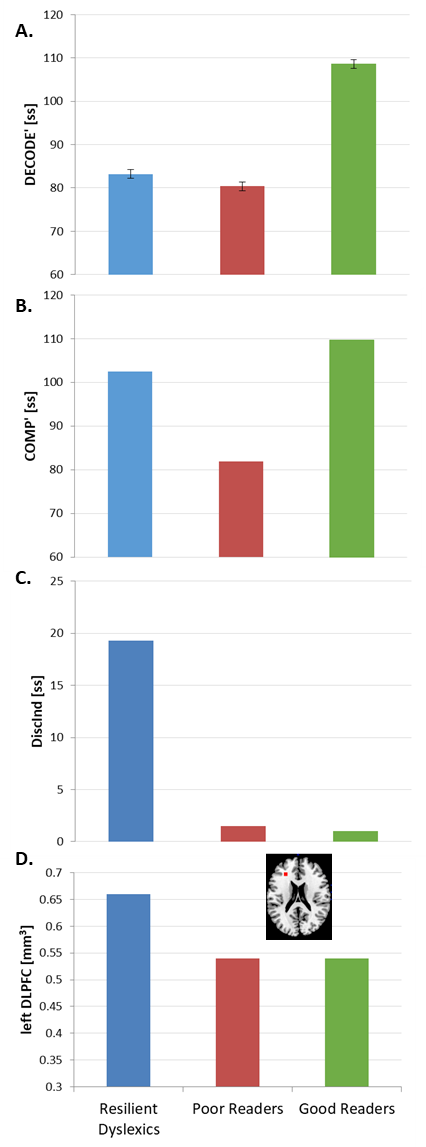
**
